# Reduced neural synchronization of gamma-band MEG oscillations in first-degree relatives of children with autism

**DOI:** 10.1186/1471-244X-8-66

**Published:** 2008-08-01

**Authors:** Donald C Rojas, Keeran Maharajh, Peter Teale, Sally J Rogers

**Affiliations:** 1 University of Colorado at Denver and Health Sciences Center, 4200 E. 9th Avenue, Denver, CO, USA; 2 The M.I.N.D. Institute, University of California at Davis, Sacramento, CA, USA

## Abstract

**Background:**

Gamma-band oscillations recorded from human electrophysiological recordings, which may be associated with perceptual binding and neuronal connectivity, have been shown to be altered in people with autism. Transient auditory gamma-band responses, however, have not yet been investigated in autism or in the first-degree relatives of persons with the autism.

**Methods:**

We measured transient evoked and induced magnetic gamma-band power and inter-trial phase-locking consistency in the magnetoencephalographic recordings of 16 parents of children with autism, 11 adults with autism and 16 control participants. Source space projection was used to separate left and right hemisphere transient gamma-band measures of power and phase-locking.

**Results:**

Induced gamma-power at 40 Hz was significantly higher in the parent and autism groups than in controls, while evoked gamma-band power was reduced compared to controls. The phase-locking factor, a measure of phase consistency of neuronal responses with external stimuli, was significantly lower in the subjects with autism and the autism parent group, potentially explaining the difference between the evoked and induced power results.

**Conclusion:**

These findings, especially in first degree relatives, suggest that gamma-band phase consistency and changes in induced versus induced power may be potentially useful endophenotypes for autism, particularly given emerging molecular mechanisms concerning the generation of gamma-band signals.

## Background

The gamma-band range of oscillatory EEG activity (30–80 Hz) has received significant attention in recent years because of its long postulated association with perceptual binding and connectivity [1,2], related concepts that have been proposed as dysfunctional in autism [3-6]. Several recent studies have reported changes in gamma-band power in people with autism from EEG or MEG recordings [7-10]. Grice et al. [8] were first to examine EEG changes in gamma in the context of face discrimination. Gamma-power was significantly greater to upright faces than to inverted faces in the healthy adult control subjects, but no differences in gamma-power between upright and inverted faces were observed in the adults with autism, despite such differences evident in the low-frequency average evoked potential. A more recent study by Brown et al. [7] studied EEG gamma-band power produced by the Kanizsa illusion (i.e., perceptual closure of incomplete geometric figures) in adolescent children with autism spectrum disorders. In this study, gamma-band power to the Kanizsa illusory shapes was reported to increase in the first 300 ms post-stimulus for the autism spectrum group, whereas a non-homogeneous comparison sample of children with "moderate learning difficulties" (i.e., IQ-matched idiopathic mental retardation) exhibited a reduction in gamma-power in the same period. Recently, Orekhova et al. [10] reported increased spontaneous EEG gamma-band power in boys with autism.

Changes in oscillatory activity such as those described for the gamma-band may be subdivided into induced changes (e.g., those which are time, but not phase-locked, to stimuli) and evoked changes [e.g., those which are both time and phase-locked to stimuli, [11]], plus gamma-band noise. Both of the visual experiments described focused on changes in induced power [7,8]. Although Brown et al. [7] do not report early phase-locked gamma results in their paper, Grice et al. [8] examined phase-locked gamma and state that no significant differences were observed between the two groups. The EEG data reported by Orekhova et al. [10] are not stimulus driven and cannot be accurately characterized as evoked or induced. We have recently reported a significant reduction in MEG-measured evoked steady-state gamma-band power in children and adolescents with autism compared to age and gender matched control subjects [9]. Using time-frequency techniques similar to those described in the current experiment, we showed that the power reduction was specific to the left hemisphere. Unlike the previous two studies, the stimulus used in that experiment (a train of clicks presented at 40/s) was designed to entrain gamma oscillatory behavior into a steady-state for the approximate duration of the stimulus (500 ms). In such stimulus conditions, very little induced gamma-power change is expected.

We undertook the current study to ascertain whether people with autism and first-degree relatives of people with autism exhibit changes in transient gamma-band power. Autism is highly heritable, with monozygotic concordance rates ranging from 36% to 91% and overall autism spectrum heritability rates estimated to be as high as 90% [12-15]. Few imaging or electrophysiological markers in probands with autism have been investigated in first-degree relatives, however. The advantage of such studies is the potential for identifying new endophenotypes of the disorder, which, in conjunction with molecular and statistical genetics techniques, could allow either for new regions of the autism genome to be identified or for stronger associations with existing gene candidates than is possible with the full and heterogeneous clinical phenotype [16,17]. In addition to examining first-degree relatives, we also employed a transient stimulus rather than a driving stimulus, so that we could examine both transient evoked and induced responses to auditory stimulation in the study. Transient, obligatory auditory gamma-band evoked responses occur within the first 100 ms after auditory stimulation [18], peaking approximately 60 ms from stimulus onset [19].

We recruited adults with autism and parents of children with autism to participate in the study and hypothesized that phase-locked auditory evoked gamma-power would be lower in the autism group and the parent group than in a healthy comparison sample, while non-phase-locked power would be significantly higher in the parent group. We further speculated that a measure of inter-trial phase-consistency for gamma oscillations, the phase-locking factor, would be reduced in the autism and parent groups relative to control subjects as a potential explanation for the proposed power changes.

## Methods

### Subjects

Nine men and seven women (mean age: 42.64 ± 5.14 years; education: 17.13 +/- 1.63 years) who are parents of a child with autistic disorder were recruited to participate in the experiment. Each parent had just one child who met DSM-IV criteria for autism, as determined by consensus of the Autism Diagnostic Observation Schedule [20], the Autism Diagnostic Interview, Revised [ADI-R: [21]] and DSM-IV diagnosis by a clinical psychologist. Nine men and 2 women with diagnoses of autistic disorder (same criteria as for the probands who qualified the parent group) participated in the study. The mean age of the autism group was 31.46 ± 9.29 years and mean years of education was 13.64 ± 1.96. All 11 participants with autism had full scale IQs greater than or equal to 70 as determined by assessment with the Wechsler Adult Intelligence Scale – 3^rd ^edition (WAIS-III: [22]). Sixteen adults (9 women) with no personal or family history of developmental disability were recruited to serve as comparison subjects (mean age: 43.14 ± 6.66 years; education: 17.25 +/- 1.61 years). All participants were tested for hearing thresholds using the method of constant stimuli and were within normal limits (< 20 dB HL) at the stimulation frequency used in the experiment. Participants signed informed consent to participate in the experiment consistent with the guidelines of the Colorado Multiple Institution Review Board.

### Stimulus delivery and MEG recordings

MEG recordings were made to monaural, contralateral ear stimulus presentations of 1 kHz sine-wave stimuli (200 ms duration, 10 ms rise/fall, 80 dB SPL at the ear). Stimuli were delivered via foam insert earphones (E.A.R., Cabot Safety Co., Indianapolis, IN). At least 150 discrete stimulus trials (4 s inter-stimulus interval) were delivered per ear.

MEG data were acquired with a 4D Neuroimaging (San Diego, CA) Magnes I neuromagnetometer system with 37 axially-wound, first-order gradiometers (5.1 cm baselines and 2 cm coil diameters). Recordings were made inside a custom-built magnetically-shielded room with 35 dB reduction at DC, and 55 dB reduction at 60 Hz. Participants were seated comfortably in a non-magnetic recording chair, and were allowed to view a silent video of their choice during recording sessions on a monitor located 4 m outside the room. Each hemisphere was recorded separately and the order of recording was random for each participant. The dewar was positioned over each side of the head so that the center channel of the hexagonal array would be centered between the minima and maxima for the M100 (approximately the T3/T4 electrode location for the 10–20 International Electrode System, or about 5 cm above the left or right fiducial reference point).

Prior to MEG recordings, the location and orientation of the MEG coils relative to each subject's head were determined by digitizing a set of fiducial reference points on the head using a magnetic digitizer (Polhemus 3SPACE). The left and right preauricular points (LPA and RPA) and the nasion, as defined in the EEG 10–20 electrode system, were used to establish a right-handed Cartesian coordinate system, where the line between LPA and RPA is the x-axis (positive x out the left ear). The y-axis is the line normal to the x-axis at the midpoint (origin), with positive y exiting through the front of the head at the nasion, and the z-axis is normal to x and y at the origin (positive z exits at the top of the head). After digitizing the reference points, the shape of each subject's head under the recording surface of the MEG system was digitized for use in constructing a volume conductor model for MEG source localizations (see below).

MEG data were digitized at 16-bits quantization at a sampling rate of 1041.7 Hz within an epoch window of 450 milliseconds (200 ms pre-stimulus and 250 ms post-stimulus) triggered by the onset of each sound. An analog pass band between 1 and 200 Hz was used during acquisition.

### Data processing and magnetic source localizations

After acquisition, all data epochs with values exceeding ± 2500 fT were rejected from further analysis to exclude trials with eye-blinks and movement artifacts. The remaining epochs were signal averaged separately for each hemisphere to produce an averaged auditory evoked magnetic field for each of the 37 sensors. Averages were then baseline corrected using the -200 to 0 ms window and were digitally band-pass filtered (24 dB/octave, phase invariant Butterworth-characteristic) at between 35 and 45 Hz for source analyses of the transient gamma-band response.

Source analyses of the averaged data were conducted using BESA 5.1 software (Megis GmbH, Germany). A best fitting conductive sphere model was constructed from the intracranial surfaces of the T1-weighted MRI scans of subjects enrolled in the study (see Peterson et al. [23] for details of the MRI acquisition and Teale et al. [24] for a description of the conductor model derivation).

**Figure 1:**
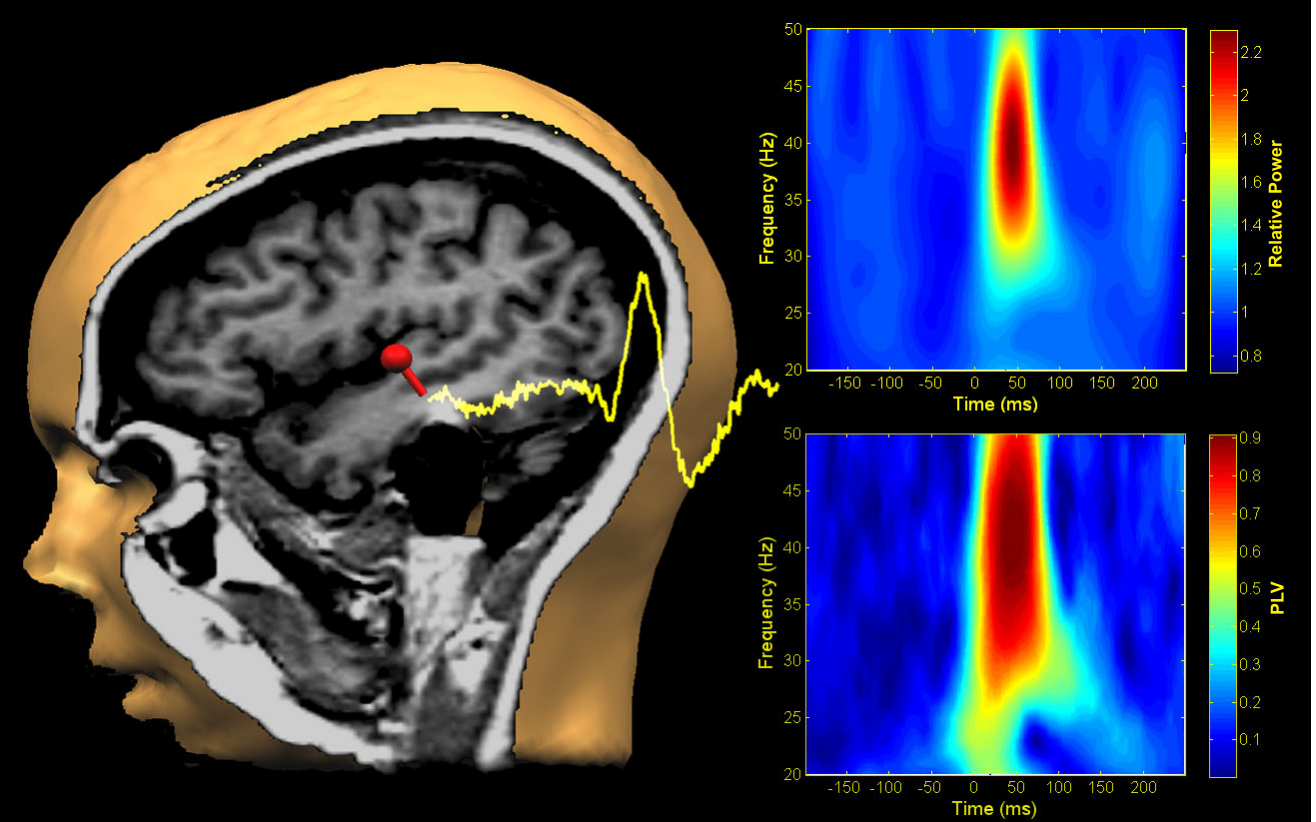
**Source space projection and time-frequency analysis.** Left hemisphere data from a single participant are illustrated. A single equivalent current dipole was fit to the sensor data and is shown overlaid onto the co-registered MRI scan for the same individual (left). The yellow waveform is the unfiltered, source-space projected, phase-locked average waveform resulting from that dipole (the M50, M100 and M200 responses can be seen in the waveform). In the upper right panel, a time-frequency plot illustrates the transient gamma-band response in terms of power relative to the pre-stimulus baseline. The lower right panel illustrates the PLF for the same data. Note the peak power and PLF centered around 50 ms post-stimulus at 40 Hz.

A single equivalent current dipole (ECD) was then fit separately for left and right hemispheres using a spatiotemporal model to the observed data in the post-stimulus window between 40–80 ms, yielding parameter estimates of the x, y, and z ECD position information, as well as dipole orientation and magnitude over time (see Figure 1). For the purpose of comparing MEG coordinates in a standardized MRI space, individual MRI scan data were spatially normalized using SPM2 (Wellcome Department of Imaging Neuroscience, London). The co-registered MEG dipoles were then transformed into Montreal Neurological Institute (MNI: [25]) space for statistical comparison.

The ECD parameters from the dipole fit were then saved and in conjunction with the individual spherical model, used to project the original raw 37-channel MEG time series into source space using source space projection (SSP, also sometimes referred to as signal space projection: [26]). SSP is an inverse-spatial filter approach that results in a significantly reduced dataset in brain, rather than sensor, space (i.e., for a single hemisphere of data, it results in 1 auditory cortex channel, or virtual electrode, rather than 37 sensor channels). Another potential advantage to SSP is that, in at least one prior paper, it produced a nearly two-fold increase in signal to noise ratio for the resulting source space measures relative to those same measures in sensor space for gamma-band power [27]). Finally, as discussed in Orekhova et al. [10], muscle artifacts are a potential challenge for intepretation of sensor-based gamma-band power measurements because of spectral overlap between muscle and brain in the gamma range. The spatial filtering approach inherent in SSP represents an efficient way to separate the signals in space rather than frequency.

The SSP channel timeseries, q(t), for the left and right hemispheres were then transformed to the frequency domain using the Morlet wavelet (wave number, 6) decomposition [28]. For each epoch, wavelet scales corresponding to frequencies from 20 to 50 Hz calculated at 1 Hz apart were used for the decomposition. For the chosen wavelet at the 40 Hz scale, the calculated temporal spreading of power with a 10 dB maximum power reduction from a test impulse response (IR) signal was approximately 40 ms. In addition, at the 40 Hz resolution, one can expect a frequency bandwidth of 13.3 Hz centered at 40 Hz [29]. Evoked (phase-locked) source strength was calculated by averaging the complex valued decomposition over trials and then taking the complex modulus (absolute value), whereas for total source strength, the complex modulus was first performed at each trial and then averaged. Induced (non phase-locked) source strength was calculated by subtracting evoked strength from the total strength [30].

In addition to evoked source strength, a more direct measure of phase consistency across trials at some specified time slice was also calculated. If there were no phase consistency in response to the stimulus across trials (at some particular time slice), then the distribution of phases would be approximately uniform. Accordingly, some dominant consistent phase angle across trials would result in a unimodal distribution. The circular variance of this distribution would then provide one method of quantifying phase consistency and is defined as [31]:

$$1-\frac{1}{n}\left(\sqrt{{\left(\sum_{i=1}^{n}\cos\theta_{i}\right)}^{2}+{\left(\sum_{i=1}^{n}\sin\theta_{i}\right)}^{2}}\right)$$

where n is the number of trials and *θ*_I _is the phase angle at the ith trial. The second term in the above equation is often described in the literature [29] as the phase locking factor (PLF) whose value spans the range from 0 to 1. A value of 1 would indicate identical phase from trial to trial. We have simulated data with ~200 trials of random phase and calculated PLFs of about 0.08. This method results in a PLF for each time point and has several benefits; 1) data are normalized which makes group averaging/comparing possible, 2) it is independent of response amplitude and 3) it is a more direct measure of the our proposed gamma-band deficit in autism.

Using these methods phase locking factor, evoked and induced source strength amplitudes over time were computed for the left and right hemispheres. The transient gamma-band window chosen for analysis consisted of the period from 40 to 80 ms post-stimulus, which encompassed the peak of the averaged transient gamma-band response for all subjects and is consistent with the peak latency of 60 ms reported in the literature [19]. Mean gamma-band evoked power, induced power and PLF in this time window were computed for statistical analysis. In addition, baseline gamma-band power was also calculated. All time-frequency computations were conducted using our own custom routines written in the MATLAB environment (MathWorks, Inc., Natick, MA). Time frequency and source location variables were statistically evaluated for group differences.

## Results

SPSS 16.0 (Chicago, IL) was used for all statistical analyses. For all ANOVA designs, type III sums of squares were used. All main effects and interaction analyses were two-tailed and conducted with a .05 alpha criterion. A priori hypotheses concerning the group factor in the ANOVA designs were evaluated as two sets of orthogonal single degree-of-freedom (df) contrasts in order to fully assess the 2 df in the group factor. All time-frequency results were produced from source space projection data.

### Time-frequency analyses

Phase-locked, or evoked 40 Hz power normalized relative to baseline power was examined using a 3 × 2 ANOVA (group by hemisphere). The group factor was contrast coded to assess the hypotheses that the controls would have significantly higher evoked power than either the autism or parent groups (Contrast 1) and to assess whether the autism group had lower power than the parent group (Contrast 2). Contrast 1 was significant, F(1,40) = 4.97, p = .03, indicating that controls had higher evoked 40 Hz power than either the autism or autism parent groups. Contrast 2 was non-significant, F(1,40) = .01, p > .05, suggesting that there were no reliable differences between the autism and autism parent group with respect to evoked power. The hemisphere and diagnosis by hemisphere interaction terms were both non-significant.

**Figure 2:**
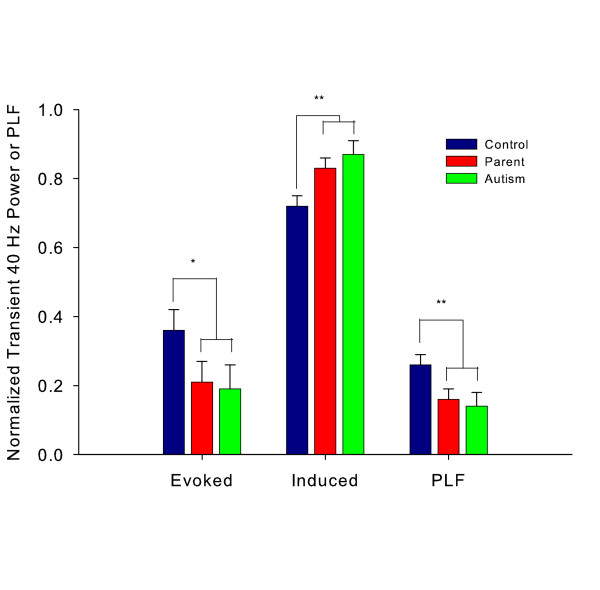
**Mean +/- SD normalized evoked and induced 40 Hz power.** The PLF data are also shown (the y-axis scale to the left is appropriate for all three measures). The lines above the bars indicate the significant comparisons for each measure (Contrast 1 in each case: see text for details). * p < .05. ** p < .01.

For induced power, the same contrasts as for evoked power were coded into the diagnosis factor in a 3 × 2 ANOVA (group by hemisphere). However, the weights were chosen to test the hypotheses that induced power would be higher in the autism and autism parent groups relative to control subjects (Contrast 1) and higher in the autism group than the parent group (Contrast 2). Induced 40 Hz power, relative to baseline, was significantly higher in the parents of children with autism and in the autism group, relative to controls (Contrast 1: F(1,40) = 10.94, p = .002). Contrast 2 was not significant, F(1,40) = 78, p > .05. The hemisphere main effect and group by hemisphere interaction term for induced power were not significant. Figure 2 illustrates the mean evoked and induced power results.

Baseline 40 Hz power was also examined in the same statistical design (3 × 2 ANOVA). We had no a priori hypotheses concerning the baseline and therefore no contrasts were employed. There was a significant main effect of hemisphere, F(1,40) = 4.56, p < .05, indicating greater baseline power in the left (27.63 +/- .87 n-Am^2^) compared to the right hemisphere (25.01 +/- 1.05 nA-m^2^). The group main effect and group by hemisphere interaction term were both non-significant.

Phase-locking factors were subjected to a 3 × 2 ANOVA, with the group factor contrast coded to assess whether the control group had higher phase-locking than either the autism or autism parent groups (Contrast 1) and whether the autism group had lower phase-locking than the autism parent group (Contrast 2). Contrast 1 was significant, F(1,40) = 8.56, p = .006 (see Figure 2). Contrast 2 was non-significant, F(1,40) = .18, p = .68. The hemisphere and group by hemisphere terms were not statistically significant.

### Source localization parameters

**Figure 3:**
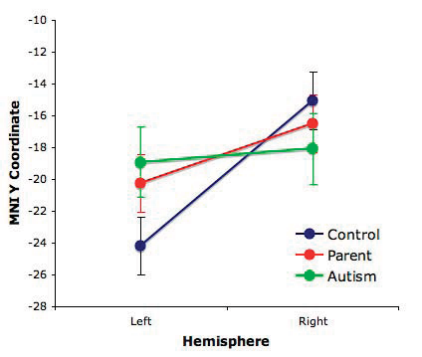
**Mean +/- SD anterior-posterior ECD coordinates.** The difference between left and right hemisphere locations is only significant for the control subjects (p < .001).

Source location parameters (x, y and z locations in MNI space, as well as x, y and z orientations and strength, in nA-m) were also subjected to statistical analysis in separate 2 × 2 mixed model ANOVAs (group by hemisphere). The mean MNI dipole locations were within the region of the left superior temporal gyrus and right Heschl's gyrus in the Automated Anatomical Labeling (AAL) template (left: x = -53.65, y = -21.37, z = 8.21; right: x = 40.02, y = -16.37, z = 13.91), which provides labels for structures in MNI space [32]. There were no group differences or group by hemisphere interactions for the x and z location parameters, the x, y, or z orientations, or the source strength, observed at .05 alpha. However, for the y-coordinate location (anterior-posterior axis), the hemisphere main effect was signicant, F(1,40) = 14.63, p < .001, indicating that the right hemisphere sources were anterior to those in the left hemisphere (left: -21.12 +/- 1.13 mm; right: -16.55 +/- 1.13 mm). A significant group by hemisphere interaction term indicated that this asymmetry differed between groups, F(1,40) = 4.12, p < .05. Post-hoc LSD testing revealed that controls had sources significantly anterior in the right (-15.06 +/- 1.82 mm) than the left hemisphere (-24.19 +/- 1.82 mm), p < .001. The anterior-posterior asymmetry of the parent group was trending to significant (left: -20.25 +/- 1.82 mm; right: -16.50 +/- 1.81 mm, p = .06), but the autism group did not exhibit any such asymmetry (left: -18.91 +/- 2.19 mm; right: -18.09 +/- 2.18 mm, p = .73, see Figure 3).

## Discussion

Induced 40 Hz gamma-band power was significantly higher in the parents of children with autism and in the autism group when compared to control participants, while evoked gamma-band power was reduced in the same comparison. Consistent with our hypotheses, the phase-locking factor, a measure of inter-trial phase consistency, was reduced in the parent and autism groups relative to control subjects. We believe that the phase-locking factor more directly reflects the underlying problem in autism (i.e., neural synchrony, or timing consistency deficits). Since total power for a particular frequency equals the phase-locked plus the non-phase-locked power (and noise), the significant increase in induced 40 Hz power accompanied by decreased evoked 40 Hz power is consistent with the significant reduction in phase-locking at 40 Hz, keeping total power constant.

Since reduced phase-locking should result in a shift of power between phase-locked (evoked) and non-phase-locked (induced) power while conserving total power, we speculate that there is no evidence for dysfunctional in autism at the level of gamma-band oscillation production in response to external stimulation; rather the dysfunction we observe in the current study is more consistent with the view that the ability to time the gamma-band power increases with external stimulation is impaired. The evoked-induced power distinction is thus more properly considered as a continuum, with timing relationships to stimuli, responses or cognitive events shifting the balance between the two extremes. This view is consistent with recent considerations of evoked and induced EEG/MEG responses [33,11]. We also propose that the increase in induced gamma-band power observed in some prior studies (e.g., [7-9]) is consistent with the decrease in evoked gamma-band power reported in our previous work [34].

It is difficult, however, to directly compare prior studies, which use stimulus-related power and/or phase measures, with those measuring spontaneous (i.e., non stimulus-driven) gamma-band power in autism. [e.g., [10]]. The literature to date appears to suggest that people with autism (and perhaps their first-degree relatives) may have higher levels of spontaneous gamma-band EEG/MEG, but that under conditions of external visual and auditory stimulation, are unable to properly time their cortical responses with the stimuli they perceive. While we found an increase in baseline total 40 Hz power in the left hemisphere relative to the right hemisphere, there was no group difference in this result. Also, under conditions of relatively constant stimulation, baseline power measures may not relate directly to spontaneous gamma-band.

The findings of this study are interesting to consider together with our recently reported findings of reduced steady-state evoked gamma-band power in children and adolescents with autism [9]. In doing so, however, several factors should be considered when comparing the two studies. First, in the previous paper, we employed children and adolescents with autism, adults with autism and adult first-degree adult relatives as in the current study. If gamma-power is partly familial, as suggested by a prior study in schizophrenia [35], then it stands to reason that dysfunctional gamma-band mechanisms will not be present in all relatives, resulting in an average difference with controls that is less than would be obtained in a comparison between probands and controls. We found, however, that while both the parent and autism groups showed reduced phase-locking and evoked power with a corresponding increase in induced power relative to controls, they did not exhibit differences from each other on any of the measures. This may either mean that the majority of the autism parents in this sample had the familial liability relevant to gamma-band disturbances or alternatively that the difference between the two groups was too small to detect with the current sample sizes. Replication with a larger sample will be necessary, as well as extension to a direct comparison between probands and their own first-degree relatives (parents and siblings).

Second, the previous study employed steady-state stimulation, which directly entrains a particular frequency (e.g., 40 Hz) in the EEG or MEG response. Our own unpublished observations suggest that this type of stimulation may accentuate group differences in gamma-power and phase-locking between groups over pure tone or unmodulated stimuli. For this study, a transient rather than driving stimulus was chosen because we were specifically interested in the obligatory, transient gamma-band response rather than the steady-state response. Although we believe that the overall result of reduced phase-locked power is consistent with the earlier proband paper, a direct comparison between probands, first-degree relatives and control subjects using both transient and steady-state stimuli will be needed to establish which stimulation method, if any, produces more robust results. A final comment regarding our earlier finding is that we did not examine either phase-locking or induced power in the previous paper. The inclusion of the adult autism sample in the current experiment would seem to suggest some continuity of the finding of reduced evoked power across two independent samples of persons with autism at much different developmental stages, but future studies should examine more thoroughly the relationships between phase-locked and non-phase-locked power in persons with autism spectrum conditions.

Considerable progress has been made recently in relating gamma-band electrophysiology to distinct cortical mechanisms. Pyramidal cell glutamatergic input to inhibitory interneurons, particularly those expressing the calcium-binding protein parvalbumin (PV), results in the recurrent and phasic inhibitory modulation of those same pyramidal cells [36-39]. PV expressing interneurons, particularly the basket cells, appear to play a critical role in this inhibitory modulation via GABA_A_-receptor mediated neurotransmission [reviewed by [39]]. The timing of the inhibitory modulation that results in gamma-band frequency output from the principal cells is thought to partly result from the inhibitory synaptic conductances of the interneuron-principal cell synapses [40].

The potential importance of GABAergic dysfunction to autism has been repeatedly stressed in the literature. [e.g., [41]]. Blatt et al. [42] reported significantly reduced GABA_A_-receptor binding in high binding regions of the hippocampus, with no significant differences noted in binding of serotonergic, cholinergic and glutamateric receptors. GABA receptor genes, most notably GABRB3 have been of significant interest recently in autism (e.g., Ma et al. [43]). Cook et al. [44] reported linkage disequilibrium between autism and a marker for GABRB3 in the 15q11-13 chromosome region, a result replicated by some studies [e.g., [45]], but not by others (e.g., [46,47]).

The reduced anterior-posterior asymmetry of the ECD coordinates for the autism and autism parent groups may be a reflection of a general neurodevelopmental disturbance in the asymmetry of the brain. Previous work in our own lab [48-51], replicated by others [52,53] has established that the dipole locations of transient evoked responses such as the M50 and M100 exhibit reduced left-right asymmetry in schizophrenia when compared to control subjects. The asymmetry reduction in schizophrenia has also been reported for other evoked responses such as the auditory, 40 Hz steady-state response [54]. Others have reported such disturbances in dyslexia [52,55] and fragile X syndrome [56]. To our knowledge, this is the first such report of reduced anterior-posterior asymmetry for the transient gamma-band response in autism. The functional significance of this hemisphere asymmetry and its reduction in any of these disorders remains unclear.

## Conclusion

The use of endophenotypes, whether behavioral or physiological, may increase the reliability of such genetic studies. The potential utility of this approach with respect to GABA was demonstrated by a study reporting that a symptom-level approach to creating family subsets based on the presence of savant skills improved genetic linkage to 15q11-13 [57]. Few studies have investigated non-invasive physiological biomarkers in first-degree relatives of persons with autism. We propose that gamma-band abnormalities such as decreased phase-locking and increased induced power, as described in this paper, may represent an easily obtained and useful endophenotype for autism research, especially given the strong mechanistic association to GABA and cortical circuitry. It should be noted, however, that studies such as this, while suggestive of familiality, do not address actual heritability of the marker. Future efforts will need to address the viability of gamma-band deficits as an endophenotype more directly through twin-studies and direct genetic association with potential candidate genes.

## Competing interests

The authors declare that they have no competing interests.

## Authors' contributions

DCR conceived the study, including the data analytic strategy and statistical analyses and drafted the manuscript. KM and PT made important contributions concerning the time-frequency analyses and source space projection approaches. SJR made a contribution to the adult autism recruitment and diagnosis as well as to the proband assessments necessary for recruiting the parent sample for the manuscript.

## Pre-publication history

The pre-publication history for this paper can be accessed here:


